# Sodium Fluctuations in Astroglia and Their Potential Impact on Astrocyte Function

**DOI:** 10.3389/fphys.2020.00871

**Published:** 2020-08-12

**Authors:** Lisa Felix, Andrea Delekate, Gabor C. Petzold, Christine R. Rose

**Affiliations:** ^1^Institute of Neurobiology, Heinrich Heine University Duesseldorf, Duesseldorf, Germany; ^2^German Center for Neurodegenerative Diseases (DZNE), Bonn, Germany; ^3^Division of Vascular Neurology, Department of Neurology, University Hospital Bonn, Bonn, Germany

**Keywords:** astrocyte, brain, sodium/potassium-ATPase, sodium imaging, sodium signaling, glutamate, gamma-aminobutyric acid, ischemia

## Abstract

Astrocytes are the main cell type responsible for the regulation of brain homeostasis, including the maintenance of ion gradients and neurotransmitter clearance. These processes are tightly coupled to changes in the intracellular sodium (Na^+^) concentration. While activation of the sodium-potassium-ATPase (NKA) in response to an elevation of extracellular K^+^ may decrease intracellular Na^+^, the cotransport of transmitters, such as glutamate, together with Na^+^ results in an increase in astrocytic Na^+^. This increase in intracellular Na^+^ can modulate, for instance, metabolic downstream pathways. Thereby, astrocytes are capable to react on a fast time scale to surrounding neuronal activity *via* intracellular Na^+^ fluctuations and adjust energy production to the demand of their environment. Beside the well-documented conventional roles of Na^+^ signaling mainly mediated through changes in its electrochemical gradient, several recent studies have identified more atypical roles for Na^+^, including protein interactions leading to changes in their biochemical activity or Na^+^-dependent regulation of gene expression. In this review, we will address both the conventional as well as the atypical functions of astrocytic Na^+^ signaling, presenting the role of transporters and channels involved and their implications for physiological processes in the central nervous system (CNS). We will also discuss how these important functions are affected under pathological conditions, including stroke and migraine. We postulate that Na^+^ is an essential player not only in the maintenance of homeostatic processes but also as a messenger for the fast communication between neurons and astrocytes, adjusting the functional properties of various cellular interaction partners to the needs of the surrounding network.

## Introduction

The maintenance of ion gradients between the cytoplasm and the extracellular space (ECS) is one of the most important functions in living cells ensuring cell survival and the execution of physiological processes. Disruptions of this homeostasis can lead to cell damage or death. Beside its importance for homeostatic processes, the transport of ions across the plasma membrane may change the membrane potential, thereby affecting cellular excitability. Changes in the ionic concentration can also modulate cellular responses, and ions themselves can act as a second messenger (Orlov and Hamet, 2006). Astrocytes, which essentially lack electrical excitability, use such ionic signaling as a main means for intracellular and intercellular communication (Verkhratsky and Nedergaard, 2018). So far, most of the studies on ionic signaling in astrocytes have focused on the role of Ca^2+^, which is nowadays widely accepted to be a critical second messenger in astroglia (Petzold and Murthy, 2011; Volterra et al., 2014; Rusakov, 2015; Verkhratsky et al., 2019).

It has become clear, however, that astrocytic ionic signaling also involves monovalent cations, among them protons, which modulate a multitude of intracellular processes (Chesler, 2003). In addition, recent work has shown that a signaling role can also be assigned to sodium ions (Na^+^), underlining the idea that Na^+^ transients may represent a new form of astrocyte excitability (Rose and Verkhratsky, 2016a). In astrocytes, changes in the intracellular Na^+^ concentration ([Na^+^]_i_) arise, e.g., in response to release of neuronal transmitters such as glutamate and gamma-aminobutyric acid (GABA; Rose and Ransom, 1996b; Chatton et al., 2000; Kirischuk et al., 2007). Astrocytic Na^+^ signaling, thereby, couples neuronal activity to fast local astrocytic responses, which, among others, include an activation of astrocytic metabolism and lactate production (Rose and Verkhratsky, 2016a; Verkhratsky et al., 2019). Spatio-temporally organized fluctuations in cytosolic Na^+^ concentration may, thus, represent a Na^+^ signaling system that coordinates astrocyte physiology, as well as glial homeostatic support, to neuronal needs. Furthermore, emerging studies in the field identified more “atypical” roles for Na^+^. These arise upon direct binding of Na^+^ to different proteins, including plasma membrane receptors and ion channels, thereby influencing a variety of cellular functions.

With this review, we aim to give an overview on the conventional Na^+^ signaling pathways in astrocytes, as well as newly identified atypical roles of Na^+^. We will start by explaining how astrocytes regulate their Na^+^ levels and then highlight the most important mechanisms involved in the generation of Na^+^ signals in astrocytes. In the following two sections, different consequences of Na^+^ signaling will be presented and their likely role in normal brain function will be discussed. The last part summarizes findings from studies that investigated how pathological changes in different diseases of the central nervous system (CNS) can affect Na^+^ signaling pathways and vice versa.

## Na^+^ Regulation in Astrocytes

### Efflux Pathways for Na^+^

**Figure 1:**
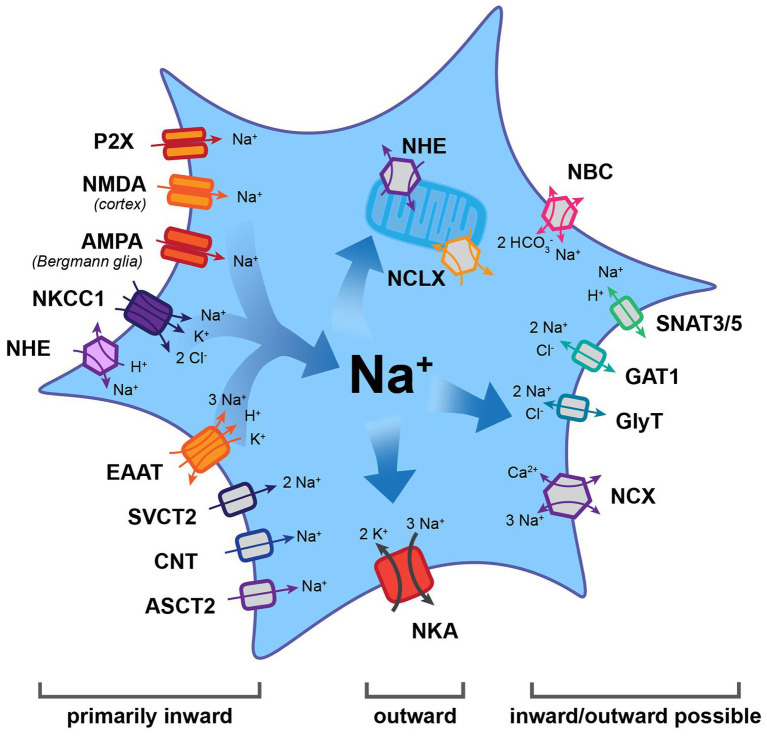
Main efflux and influx pathways for Na^+^ in astrocytes. Note that the Na^+^/K^+^-ATPase (NKA) is the major mechanism for Na^+^ export. Other transporters may function in the inward or outward mode, thereby generating influx or efflux of Na^+^. Most other carriers and all ion channels mediate Na^+^ influx into astrocytes. AMPA, α-amino-3-hydroxy-5-methyl-4-isoxazolepropionic acid receptor; ASCT2, alanine, serine, cysteine transporter 2; CNT, concentrative nucleoside transporter; EAAT, excitatory amino acid transporter; GAT, gamma-aminobutyric acid transporter; GlyT, glycine transporter; NBC, Na^+^-HCO_3_^−^ cotransporter; NCX, Na^+^/Ca^2+^ exchanger; NCLX, mitochondrial Na^+^/Ca^2+^/Li^+^ exchanger; NHE, Na^+^/H^+^ exchanger; NKA, Na^+^/K^+^-ATPase; NKCC1, Na^+^-K^+^-2 Cl^−^ co-transporter 1; NMDA, N-methyl-d-aspartate receptor; SNAT3/5, sodium-coupled neutral amino acid transporter; and SVCT2, sodium-dependent vitamin C transporter 2.

Astrocytes rely on a steeply inwardly directed Na^+^ gradient to drive a multitude of membrane transport processes. The gradient is based on their low [Na^+^]_i_ reported values, for which vary between 10 and 17 mM (Kirischuk et al., 2012; Rose and Karus, 2013; Felix et al., 2020), coupled with an extracellular Na^+^ concentration ([Na^+^]_o_) of 140–150 mM (Rose and Karus, 2013). The primary mechanism for its installation is the sodium-potassium-ATPase (NKA), which uses energy obtained *via* ATP breakdown in order to transport 3 Na^+^ and 2 K^+^ ions against their concentration gradients (out of and into the cell respectively; Jorgensen et al., 2003; Aperia, 2007; Hertz et al., 2015; Figure 1). The expression of the NKA is among the highest of all proteins in astrocytes, with around 90% of the isoforms being α2β2 and the final 10% being α2β1 (Stoica et al., 2017). NKA activity can be dampened by ouabain-like molecules, which exist endogenously within the brain and primarily target the α subunit (Song and Du, 2014). This effect is mimicked by the external application of ouabain, which – when applied at fully-blocking concentrations – causes a massive increase in [Na^+^]_i_ (Kimelberg et al., 1979; Rose and Ransom, 1996a; Chatton et al., 2000).

While NKA constitutes the major pathway for Na^+^ efflux, it is not the only way to export Na^+^. A number of transporters, for which the “forward” mode involves Na^+^ influx, have reversal potentials (E_rev_) that lie close to the highly negative resting membrane potential of astrocytes. Depending on the ion gradients and membrane potential, they may reverse and, therefore, offer alternative pathways for Na^+^ removal. The membrane potential of astrocytes is mainly set by expression of different K^+^ channels, among them Kir4.1 and K_2_P, that allow the efflux of K^+^ (Verkhratsky and Nedergaard, 2018). Notably, the values established for astrocytes’ resting membrane potential differ between different conditions and preparations. While initial studies reported membrane potentials of around −70 to −80 mV (e. g., Wallraff et al., 2006; Zhou et al., 2006), other studies determined even higher values (e.g., −83 mV: Amzica et al., 2002; −85 mV: Kafitz et al., 2008; or −87 mV: Mishima and Hirase, 2010). A correct prediction of the transport direction of “readily-reversible” transporters, thus, requires not only knowledge of the ion gradients of the transported ions but also of the exact membrane potential of the astrocyte studied. Individual astrocytes in the intact tissue, however, seem to be protected from large changes in their membrane potential because the electrical coupling provided by gap junctions rapidly equalizes membrane potentials in the syncytium (Ma et al., 2016).

Among the transporters known to reverse under physiological conditions is the Na^+^/Ca^2+^ exchanger (NCX; Figure 1), of which three subtypes t (1, 2, 3) have been reported in astrocytes (Rose et al., 2020). NCX exchanges 3 Na^+^ for 1 Ca^2+^ and its E_rev_ lies between −90 and −60 mV; it can therefore operate either in forward (Na^+^ in) or reverse (Na^+^ out) mode (Verkhratsky et al., 2018; Rose et al., 2020). The latter is also true for the Na^+^-HCO_3_^−^ cotransporter (NBC; Figure 1), which serves to regulate HCO_3_^−^ (and pH) in astrocytes (Chesler, 2003). Gray matter astrocytic NBCe1 transport 2 HCO_3_^−^ for every Na^+^, resulting in an E_rev_ of about −80 mV (Chesler, 2003). This makes the cotransporter easily reversible, offering a further mechanism to export Na^+^ (Theparambil et al., 2014).

Transport reversal, involving the efflux of Na^+^ under close-to-physiological conditions, was also demonstrated for several transporters for uptake of transmitters. These include gamma-aminobutyric acid transporters (GATs) and glycine transporters (GlyTs), which both transport 2 Na^+^ and 1 Cl^−^ along with one GABA or glycine molecule, respectively (Eulenburg and Gomeza, 2010; Zhou and Danbolt, 2013; Figure 1). These electrogenic systems reverse at around −80 to −70 mV. In addition to depolarization, reversal can be triggered by increases in [Na^+^]_i_, e.g., following activation of glutamate uptake by excitatory amino acid transporters (EAATs; Huang and Bergles, 2004; Shibasaki et al., 2017). Astrocytic transport of glutamine across the membrane is mediated by sodium-coupled neutral amino acid transporters 3 and 5, SNAT3/5 (Figure 1), cotransporting 1 Na^+^ and countertransporting a proton (Deitmer et al., 2003; Uwechue et al., 2012). Glutamine export is realized in the “outward” mode – which means exporting Na^+^ – and constitutes a critical component of the glutamate-glutamine cycle (Danbolt, 2001).

Localized export of Na^+^ from the cytosol can additionally be managed *via* its uptake into mitochondria by Na^+^/Ca^2+^/Li^+^ exchangers (NCLX; Figure 1; Palty et al., 2010; Verkhratsky et al., 2018). These take in 3 Na^+^ while releasing 1 Ca^2+^ from the organelle and their activity is highly sensitive to activity-induced [Na^+^]_i_ changes (Maack et al., 2006; Parnis et al., 2013). NCLX action has important implications, as it alters available Ca^2+^ in the mitochondria and the cytosol, while allowing mitochondria to act as a kind of Na^+^ store (Ben-Kasus Nissim et al., 2017). In line with this view, mitochondrial [Na^+^] in astrocytes has been shown to be higher than the surrounding cytosol (Bernardinelli et al., 2006; Meyer et al., 2019). The release of Na^+^ from these stores appears to be primarily mediated by the mitochondrial Na^+^/H^+^ exchanger (NHE; Bernardinelli et al., 2006).

### Na^+^ Influx Pathways

One of the best-described functions of astrocytes is their uptake of transmitters from the extracellular space. They carry this task out *via* transporters, which typically bring Na^+^ into the cell, and use the energy gained to take in specific signaling molecules. Among these is glutamate, which is taken up by the EAATs glutamate-aspartate transporter (GLAST) and glutamate transporter 1 (GLT-1; Rothstein et al., 1996; Danbolt, 2001; Figure 1). EAATs bring in one glutamate along with a proton using the energy gained from importing 3 Na^+^, while simultaneously exporting 1 K^+^. Their E_rev_ lies at around +50 mV, making reversal extremely unlikely (Eulenburg and Gomeza, 2010; Zhou and Danbolt, 2013). Similarly, transporters for ascorbic acid (sodium-dependent vitamin C transporter 2, SVCT2), adenosine (concentrative nucleoside transporter, CNT), and D-serine (alanine, serine, cysteine transporter 2, ASCT2) all have an E_rev_ of around +60 mV (Figure 1). While CNT2 and ASCT2 have a stoichiometry of 1 Na^+^: one substrate, the SVCT2 brings in 2 Na^+^ for every ascorbic acid molecule (Siushansian et al., 1997; Acuna et al., 2013; Maucler et al., 2013; Martineau et al., 2014; Rose and Verkhratsky, 2016b).

In addition, astrocytes heterogeneously express ionotropic transmitter receptors, which can trigger influx of Na^+^. For example, cortical but not hippocampal, astrocytes express N-methyl-d-aspartate (NMDA) receptors (Schipke et al., 2001; Lalo et al., 2006) and Bergmann glial cells express α-amino-3-hydroxy-5-methyl-4-isoxazolepropionic acid (AMPA) receptors (Matsui, 2005). Furthermore, there is evidence for astrocytic expression of purinergic P2X receptors (Figure 1), which – depending on their subunit composition – may produce and inward Na^+^ current (Lalo et al., 2008; Verkhratsky et al., 2012).

Prominent influx of Na^+^ into astrocytes is provided by the Na^+^/H^+^ exchanger NHE1 (Figure 1), which plays a central role in their pH regulation (Chesler, 2003). It has a very positive E_rev_ (60–70 mV) and permanently functions in the forward mode under physiological conditions – taking in 1 Na^+^ and extruding 1 proton (Chesler, 2003; Rose and Verkhratsky, 2016b). Like NHE1, the Na^+^-K^+^-2 Cl^−^ (NKCC1) cotransporter (Figure 1) is electro-neutral, importing 1 Na^+^ along with 1 K^+^ and 2 Cl^−^. The result of that is that its operation at rest is inward under physiological conditions, with reversal only possible after extensive increases in [Na^+^]_i_ during pathological conditions (Su et al., 2002; MacAulay and Zeuthen, 2012).

Many more proteins do mediate Na^+^ import into astrocytes and the reader is referred to several recent reviews presenting these in detail (e.g., Verkhratsky et al., 2019). What is clear is that inward transport of Na^+^ along its electrochemical gradient offers a reliable mechanism for the regulation of other ions and diverse substances. Furthermore, the resulting changes in astrocytic [Na^+^]_i_ can themselves allow astrocytes to respond to changes in their environment by triggering other pathways as detailed in section “Functional Consequences of Astrocytic Na^+^ Signaling in the Healthy Brain.”

## Na^+^ Signaling in Astrocytes

### Spontaneous Na^+^ Fluctuations

In addition to responding to neuronal activity, astrocytes are capable of communication within their own network, also independently from neurons. This had long been described in terms of spontaneous Ca^2+^ signals, which are present in astrocytes across several brain regions and developmental stages (Aguado et al., 2002). Seemingly spontaneous ion signaling in astrocytes, is, however, not restricted to Ca^2+^. In addition, spontaneous Na^+^ transients were reported from mitochondria of cultured astrocytes. These were around 10 s long and ~35 mM in amplitude and could be inhibited by NHE antagonists (Azarias and Chatton, 2011).

**Figure 2:**
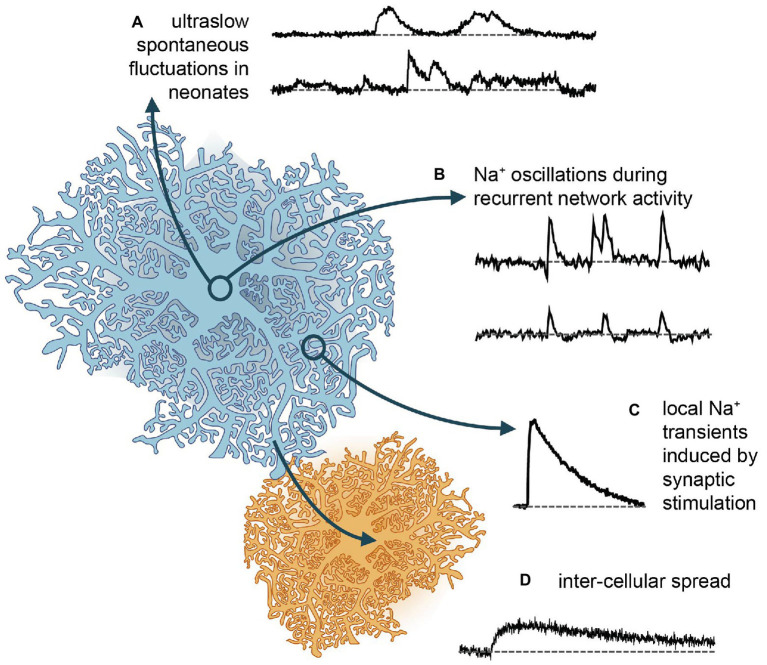
Different forms of Na^+^ signaling in astrocytes. **(A)** Spontaneous Na^+^ fluctuations as recently reported from neonate hippocampus and cortex (Felix et al., 2020). **(B)** More rapid, global Na^+^ oscillations, which are synchronized between cells and accompany recurrent network activity in mouse hippocampus (Karus et al., 2015, 2017). **(C)** Local Na^+^ transients induced in different types of astrocytes and brain regions upon afferent synaptic stimulation of glutamatergic fibers (e.g., Kirischuk et al., 2007; Langer and Rose, 2009). **(D)** Gap junction mediated rapid spread of Na^+^ signals in the astrocytic syncytium to neighboring cells, which then depict slower kinetics and reduced amplitudes (Langer et al., 2012, 2017).

A recent study showed that neonatal astrocytes of mouse hippocampus and cortex display extremely long (~8 min), asynchronous and irregular changes in their somatic [Na^+^], termed “ultraslow Na^+^ fluctuations” (Felix et al., 2020; Figure 2). These events had amplitudes of ~2 mM and were largely limited to the first 2 postnatal weeks. Comparable Na^+^ fluctuations were measured in neurons of the same developmental stage, and these could be attributed to GABAergic signaling. However, antagonists for GABAergic components had no effect on the astrocytic signals – as was the case with several other major pathways (glutamate, acetylcholine, nor-adrenaline, and glycine). Astrocytic signals were dampened by the application of tetrodotoxin to block voltage-gated Na^+^ channels. While this linked them to neuronal activity, it remained unclear which pathways are involved in their generation (Felix et al., 2020). Independent of the – as yet – unknown origin of the neonate ultraslow Na^+^ fluctuations, the timing of their appearance makes it tempting to assign them a developmental function, as has been shown to be the case for other spontaneous activity patterns in the young brain (Ben-Ari, 2001).

### Na^+^ Transients Evoked by Neuronal Activity

#### Na^+^ Transients in Cultured Cells

The first Na^+^ signals measured in astrocytes were recorded in cell culture. Here, neuronal activity can be mimicked and Na^+^ transients evoked by direct application of transmitters. Under these conditions, glutamate or its agonists evoke [Na^+^]_i_ increases of up to 20–30 mM in hippocampal astrocytes (Rose and Ransom, 1996a). The transients take up to a minute to reach their peak and typically return to their baseline levels within several minutes. Antagonist applications revealed these elevations to be primarily caused by EAAT-mediated glutamate uptake, with minor roles for ionotropic receptors (Rose and Ransom, 1996a; Chatton et al., 2000). Application of GABA under comparable conditions produces Na^+^ transients with a similar time scale, albeit with a smaller amplitude of ~7 mM (Chatton et al., 2003; Unichenko et al., 2012).

#### Na^+^ Transients in Astrocytes *in situ*

Astrocytic Na^+^ responses occur in brain tissue slices as well. Notably, the properties of these responses and the mechanisms underlying them are heterogeneous across brain regions. In cerebellar Bergman glial cells, application of glutamate induced [Na^+^]_i_ increases in the range from 2 to 20 mM (Kirischuk et al., 1997, 2007; Bennay et al., 2008). These transients are faster than those reported from cell culture, with a rise time of several seconds and a mono-exponential decay time constant of ~25 s at room temperature. Transients recovered twice as fast at 32–34°C, indicating a major role of NKA in Na^+^ export (Bennay et al., 2008). Pharmacological blockers confirmed that most of the response could be replicated by activation of EAATs alone. However, smaller transients could be induced both by applications of AMPA or kainate (but not NMDA), confirming functional AMPA receptor expression in Bergmann glia (Kirischuk et al., 2007; Bennay et al., 2008). Stimulation of either parallel and climbing fibers produced [Na^+^]_i_ increases with rise and decay times of 5 and 90 s respectively, and amplitudes of ~9 mM (Bennay et al., 2008; Figure 2). While climbing fiber stimulation resulted in global [Na^+^]_i_ increases, with a similar amplitude and time course across all processes, parallel fiber stimulation induced large Na^+^ transients near the point of stimulation, which declined with increasing distance, indicative of the pattern of activated synapses (Bennay et al., 2008).

In contrast to Bergmann glia, the ionotropic receptor component of Na^+^ transients in astrocytes within the hippocampus and Calyx of Held is minimal. These both react to puff application of d-aspartate (which activates EAATs) with [Na^+^]_i_ increases of around 5 mM (Langer and Rose, 2009; Uwechue et al., 2012). Comparable transients are induced in hippocampal astrocyte processes by Schaffer collateral stimulation (Langer and Rose, 2009). Stimulation with high intensity, presumably activating many afferent fibers, create large increases in [Na^+^]_i_, which can spread throughout the whole cell and further to adjacent cells (Figure 2). Stimulation with low intensity, in contrast, creates localized rises in [Na^+^]_i_ in individual processes, presumably nearest the synaptic release sites (Langer and Rose, 2009; Langer et al., 2017). Global, network-wide Na^+^ oscillations are induced by hippocampal network activity during neuronal “disinhibition” (Karus et al., 2015; Figure 2). Herein, blocking (inhibitory) GABA_A_-receptor activation and omission of Mg^2+^ in the perfusate produce epileptiform bursting. Under these conditions, hippocampal astrocytes undergo repetitive, synchronized increases in their [Na^+^]_i_, with amplitudes as large as 8–9 mM, followed by a brief undershoot in a subset of cells (Karus et al., 2015; Figure 2).

It is again worth to note that neocortical astrocyte Na^+^ transients have properties which distinguish them from astrocytes of the hippocampus proper, as cortical cells express functional NMDA receptors (Schipke et al., 2001; Lalo et al., 2006). In layer 2/3 astrocytes, application of glutamate or synaptic stimulation produces Na^+^ transients with an amplitude twice as large as those induced by the same protocol in hippocampal astrocytes (Ziemens et al., 2019). Large [Na^+^]_i_ increases of ~13 mM were induced specifically in processes and were found to pass to the soma. Application of AP5 (an NMDA receptor antagonist) reduces these signals by 50%, and further addition of TFB-TBOA (to block EAAT responses) eliminates the transients completely. AMPA receptors appear not to contribute to astrocyte Na^+^ signals in the neocortex (Ziemens et al., 2019).

Na^+^ transients are not limited to gray matter. Astrocytes in the corpus callosum respond to glutamate application and electric stimulation with increases of around 2–5 mM and 1–2 mM, respectively. As is the case for protoplasmic astrocytes of the hippocampus – the majority of the measured Na^+^ transients are removed by the application of EAAT blockers, suggesting that here too, glutamate uptake is the primary pathway for Na^+^ influx (Moshrefi-Ravasdjani et al., 2017, 2018).

#### Na^+^ Microdomains and Diffusion

A special compartment allowing localized increases in astrocytic [Na^+^]_i_ was postulated by Blaustein et al. (2002). In analogy to work by the Lederer et al. (1990), they proposed that the presence of the endoplasmic reticulum (ER) very close to the membrane creates a confined area, wherein Na^+^ elevations could reach concentrations high enough to reverse the NCX – and thereby induce further Ca^2+^ release from intracellular stores. Immunohistochemical work indeed provided evidence that both the NCX and the α2β2 form of NKA are clustered around areas where the ER lies near the plasma membrane in astrocytes (Blaustein et al., 2002; Lee et al., 2006). Conversely, other work showed no evidence for such Na^+^ domains at the ER adjacent sites, and the concept remains controversial (Lu and Hilgemann, 2017; Sachse et al., 2017).

Notably, the idea of ionic signaling microdomains to be present in astrocytes was again promoted by recent modeling work. Here, the authors proposed that the flow of cations in small perisynaptic processes enwrapping synapses like a “cradle” is strongly restricted, giving rise to astrocytic K^+^ and Na^+^ microdomains close to synapses (Breslin et al., 2018). In a follow-up paper, this conception was explored further to demonstrate that local increases in [Na^+^]_i_ promote a local reversal of the NCX (Wade et al., 2019). Very small compartments may, thus, form subcellular Na^+^ domains, also with different resting concentrations to the rest of the cell, which could in turn impact the direction of the transporter function in these areas.

The extent to which Na^+^ transients can spread throughout astrocytes is critical also because changes in [Na^+^]_i_ can directly pass on to mitochondria. *In vitro* applications of agonists including glutamate, kainate, d-aspartate, or AMPA, all showed mitochondrial [Na^+^] rises to a similar extent as their surroundings, for the duration of application (Bernardinelli et al., 2006). Although the function of this increase remains unclear, its recruitment of the NCLX and NHE is likely to impact respiratory pathways by altering the availability of both Ca^2+^ and protons (Azarias and Chatton, 2011).

While local microdomains established by fine perisynaptic processes may strongly restrict the diffusion of Na^+^ in astrocytes, Na^+^ diffusion along larger processes (large meaning microscopic scale) is very rapid in astrocytes, reaching velocities of >100 μm/s (Langer et al., 2012, 2017). Na^+^ can moreover rapidly pass through gap junctions into neighboring cells – across the astrocytic syncytium (Langer et al., 2012, 2017; Figure 2). This spread has been shown to extend to other cell types with astrocytes, oligodendrocytes, and NG2 glia being connected into a panglial network in both gray, and white matter (Griemsmann et al., 2015; Augustin et al., 2016; Moshrefi-Ravasdjani et al., 2017, 2018). The spreading of Na^+^ in this manner may help to maintain the low [Na^+^]_i_ critical for driving uptake at/near the site of its influx.

Spread of Na^+^ from cells with locally elevated [Na^+^]_i_ toward cells with a lower [Na^+^]_i_ will not only be driven by the concentration gradient but also be dependent on the membrane potential of the involved cells. While the isopotentiality of the gap-junction-coupled astrocyte network might be initially be disturbed at the site of Na^+^ influx, it will effectively dampen resulting changes in the membrane potential and thereby also facilitate the intercellular spread of Na^+^, as has been shown for redistribution of K^+^ (Ma et al., 2016).

## Functional Consequences of Astrocytic Na^+^ Signaling in the Healthy Brain

**Figure 3:**
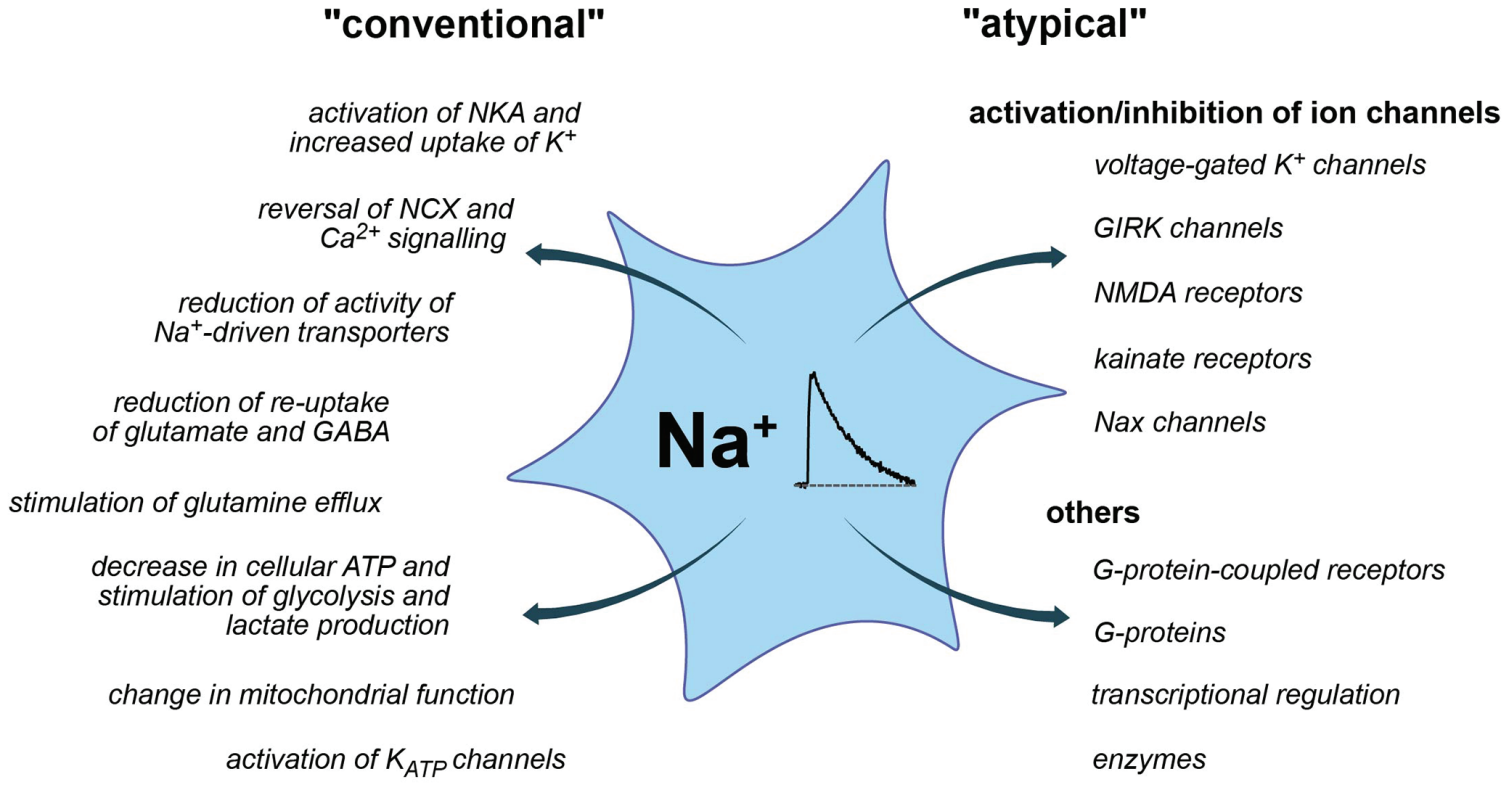
Conventional and more “atypical” consequences of Na^+^ and Na^+^ signaling. **Left:** conventional roles, largely established and accepted to be relevant for astrocytes. **Right:** atypical roles, partly hypothetic and based on insights from other systems.

So far, most studies addressed the possible consequences of changes in astrocytic Na^+^ in the context of altered driving force for Na^+^ dependent transporters. The latter couple Na^+^ homeostasis to the regulation of other ions, neurotransmitters, and diverse substrates processes not only highly relevant for astrocytic function but also for their communication with neurons (Kirischuk et al., 2012; Rose and Karus, 2013; Verkhratsky et al., 2019). Increases in [Na^+^]_i_ moreover involve its re-export through the NKA, thereby coupling Na^+^ signaling to astrocyte metabolism (Pellerin and Magistretti, 2012). In addition, Na^+^ also exerts “atypical” roles (Figure 3), which are not related to changes in driving forces nor directly change the cellular energy status. Instead, a binding of Na^+^ from either the intracellular or extracellular side, directly effects the functionality of discrete proteins as discussed below.

### “Conventional” Roles

#### Na^+^ and Regulation/Signaling of Other Ions

##### NKA and K^+^ Clearance

It has long been established that the pump cycle of the NKA is dependent on binding of Na^+^; once all three binding sites for Na^+^ are occupied, the transporter undergoes conformational changes that regulate the cleavage of ATP (Clausen et al., 2017). In addition, mainly based on work on cardiac myocytes, a secondary activation of NKA by Na^+^ was proposed, complemented by a so-called “Na^+^-deficiency inactivation” that may serve to maintain the pumps in a “reserve state” when not needed (Hilgemann, 2020).

Astrocytic NKA plays an important role in the regulation of extracellular K^+^ concentration ([K^+^]_o_) (Figure 3). Owing to its predominant subunit composition (α2/β2), it is selectively stimulated by increases in [K^+^]_o_, which triggers K^+^ uptake into astrocytes (Karus et al., 2015; Larsen et al., 2016). K^+^-induced activation of astrocytic NKA can in fact result in a decrease in [Na^+^]_i_ below baseline (Rose and Ransom, 1996a; Karus et al., 2015). The minority isoform α2β1 is insensitive to changes in [K^+^]_o_ and its activity can instead be increased by elevations in astrocytic [Na^+^]_i_ (Larsen et al., 2016). In neurons, NKA is mainly activated by increases in [Na^+^]_i_, which contributes to their repolarization following high-frequency bursts of action potentials and may induce a long-lasting post-tetanic hyperpolarization (e.g., Gulledge et al., 2013). In *Drosophila* motor neurons, the NKA-mediated afterhyperpolarisation was suggested to serve as a specific form of long-term memory of recent activity (Glanzman, 2010; Pulver and Griffith, 2010). In astrocytes, where changes in the membrane potential play a less prominent role, such effects were not yet described. Notably, the K^+^-induced activation of NKA and accompanying decrease in astrocytic [Na^+^]_i_, as well as efflux of positive charge are often counteracted or completely masked by Na^+^ import *via* glutamate transporters (Langer and Rose, 2009; Karus et al., 2015) and/or by GABA uptake (Chatton et al., 2003; Unichenko et al., 2013). The resulting increases in astrocytic [Na^+^]_i_ then again will further promote NKA activity (and K^+^ uptake).

##### NKCC1 and Cl^−^ Homeostasis

Another Na^+^-dependent transporter for uptake of K^+^ into astrocytes is the NKCC1. In cultured astrocytes and Bergmann glial cells in brain slices, NKCC1 is constitutively active, providing constant influx of all three ions (Rose and Ransom, 1996a; Untiet et al., 2017; Noor et al., 2018). Owing to its apparent low affinity for K^+^, NKCC1 only effectively contributes to clearance of extracellular K^+^ with increases above the ceiling level of 10–12 mM (Lenart et al., 2004; Hertz et al., 2015; Larsen et al., 2016). In addition, NKCC1 plays a major role in Cl^−^-homeostasis of astrocytes. In Bergmann glial cells, NKCC1 is responsible for its active accumulation, generating an outwardly-directed Cl^−^ gradient (Untiet et al., 2017). This in turn mediates the depolarizing effect of GABA_A_ receptor activation on astrocytes (Kettenmann et al., 1984; MacVicar et al., 1989). Increases in astrocytic [Na^+^]_i_, reducing the driving force for NKCC1, might therefore, lower astrocyte Cl^−^ concentrations. This will reduce the “excitatory” effect of GABA on astrocytes, also dampening associated Ca^2+^ transients (Fraser et al., 1995; Meier et al., 2008).

##### pH Homeostasis

pH regulation in astrocytes by NHE and NBCe1 is intimately related to their Na^+^ homeostasis (Deitmer and Rose, 1996; Chesler, 2003). Given, the very positive E_rev_ of the NHE (> +60 mV), proton export will not be significantly hampered unless [Na^+^]_i_ is elevated strongly. This is in stark contrast to NBCe1. Owing to its reversal potential of approximately −80 mV, a rise in astrocytic [Na^+^]_i_, together with an intracellular alkalosis, will dampen inward NBC activity and with strong Na^+^ loading and/or alkalosis, NBC may even reverse (Theparambil et al., 2015). In the brain stem, Na^+^ uptake by astrocytes is central for CO_2_/H^+^-sensing (Turovsky et al., 2016): a physiological increase in P_CO2_ activates inwardly-directed NBCe1, increasing astrocytic [Na^+^]_i_. This triggers reversal of the NCX, astrocytic Ca^2+^-signaling, release of ATP, and adaptive responses in neighboring neurons of the respiratory network (Turovsky et al., 2016).

##### NCX and Ca^2+^-Signaling

Na^+^ elevations in astrocytes that are large enough to trigger secondary Ca^2+^ signaling through NCX can arise after influx through Na^+^-permeable ion channels (Pappalardo et al., 2016; Verkhratsky et al., 2018; Rose et al., 2020). This was shown, e.g., for neocortical astrocytes upon activation of NMDA receptors (Ziemens et al., 2019). The resulting NCX-related Ca^2+^ import added to the influx through the receptor itself and significantly prolonged the duration of Ca^2+^ signals. In neural (radial glia like) stem cells of the subependymal zone, Na^+^ influx through epithelial sodium channels (ENaC) stimulated Ca^2+^ signaling, presumably through reverse NCX, contributing to the regulation of their proliferation (Petrik et al., 2018). Of note, and similar to Na^+^-dependent regulation of NKA activity described above, NCX was proposed to be subject to a secondary Na^+^-dependent inactivation following Na^+^ binding to its inward facing transport sites in cardiac myocytes (Hilgemann, 2020). Such Na^+^-dependent inactivation of NCX in astrocytes could reduce Ca^2+^ influx upon its reversal, a process which might be relevant to dampen Ca^2+^ overload under pathological conditions.

In addition to be aforementioned studies, there is an overwhelming number of other reports demonstrating Na^+^-driven reversal of astrocytic NCX. By coupling Na^+^ transients to Ca^2+^-signaling, NCX represents a direct link between those two ions and forms of astrocytic excitability. Experimental evidence supporting the existence of this important link was reviewed recently and we kindly refer the reader to this earlier work (Kirischuk et al., 2012; Verkhratsky et al., 2019; Rose et al., 2020).

#### Na^+^-Dependent Regulation of Transmitter Levels

The Na^+^ gradient of astrocytes is intimately linked to their uptake of transmitters and regulation of extracellular transmitter levels (Verkhratsky et al., 2019). Again, there are relevant conceptual differences in the biophysical properties of the transporters involved. The stoichiometry of some transporters enables reversal under severe pathological conditions only, that is, with very strong increases in [Na^+^]_i_. Among those are GLAST and GLT-1, which operate as importers for glutamate over a wide range of physiological states but can release it in the core region of an ischemic stroke where [Na^+^]_i_ may rise to >50 mM (Friedman and Haddad, 1994; Rossi et al., 2000). Other transmitter transporters (e.g., those for GABA) operate close to their reversal potential, apparently enabling both uptake and release of their substrate, depending on the conditions (see section “Efflux Pathways for Na^+^”).

Glutamate uptake is one of the major pathways for generation of astrocytic Na^+^ signaling (Rose and Verkhratsky, 2016b). Related [Na^+^]_i_ increases in the bulk cytosol are, however, apparently dampened by co-localization of GLAST and GLT-1 with α2-containing NKA (Cholet et al., 2002; Rose et al., 2009; Bauer et al., 2012; Illarionava et al., 2014; Melone et al., 2019). At the same time, even moderate glutamate-induced Na^+^ signaling can cause reversal of the NCX and thereby induce Ca^2+^ signals (Rojas et al., 2007; Rose et al., 2020). Na^+^-triggered, NCX-mediated Ca^2+^-signaling has been implemented in the mobility of mitochondria, coupling neuronal activity to astrocyte metabolism (Jackson and Robinson, 2018). Since both GLAST and GLT-1 activity are mainly energized by the Na^+^ gradient, increases in [Na^+^]_i_ reduce their driving force and may exert a negative feedback on extracellular glutamate clearance (Barbour et al., 1991; Bergles et al., 2002; Kelly et al., 2009; Unichenko et al., 2012). Notably, [Na^+^]_i_ elevations in astrocytes also drive the efflux of glutamine through Na^+^-dependent system N transporters (Broer and Brookes, 2001; Todd et al., 2017), thereby enabling its direct re-supply to neurons (Uwechue et al., 2012). Na^+^ signals thus directly link extracellular glutamate clearance to the re-cycling of its precursor glutamine to neurons.

As opposed to GLAST and GLT-1, the transporters for uptake of GABA and glycine can reverse much more easily (Eulenburg and Gomeza, 2010). Astrocytes may, therefore, not only serve to clear the extracellular space (ECS) from these inhibitory transmitters but also function as their source, depending on the prevailing driving forces (Kirischuk et al., 2016). While GABA uptake is linked with the uptake of 2 Na^+^ only, its application still results in well-detectable Na^+^ signals in astrocytes (Chatton et al., 2003; Doengi et al., 2009; Unichenko et al., 2012; Boddum et al., 2016). A reversal of astrocytic GABA transporters, presumably triggered by the related increase in [Na^+^]_i_, was demonstrated upon stimulation with glutamate (Heja et al., 2009; Unichenko et al., 2013). In hippocampal astrocytes, GAT-induced increases in [Na^+^]_i_ caused reversal of NCX, Ca^2+^ signaling, and release of ATP/adenosine, which inhibited presynaptic release of glutamate from neighboring neurons (Boddum et al., 2016). This again exemplifies the tight coupling of astrocyte [Na^+^]_i_ increases with functionally relevant Ca^2+^-signaling.

#### Na^+^-Dependent Regulation of Cellular Metabolism

Influx of Na^+^ into astrocytes necessitates its export through the NKA, increasing ATP consumption and resulting in a decrease in ATP levels (Chatton et al., 2000; Langer et al., 2017; Winkler et al., 2017; Lerchundi et al., 2019). These triggers increased uptake of glucose, enhances the breakdown of glycogen, and stimulates glycolysis, as well as the production of lactate (Brown and Ransom, 2007; Pellerin and Magistretti, 2012). The latter is assumed to play a key role in the neuro-metabolic coupling between neurons and astrocytes (Chatton et al., 2016; Magistretti and Allaman, 2018).

As mentioned above, cytosolic Na^+^ transients can be transmitted to mitochondria (Bernardinelli et al., 2006). Moreover, mitochondria may also generate seemingly spontaneous transient elevation in their [Na^+^]_i_ (Azarias and Chatton, 2011). While the functional consequences of such mitochondrial Na^+^ signaling in astrocytes are not understood yet, they might change the driving force of the mitochondrial Na^+^/Ca^2+^ exchanger NLCX, influencing mitochondrial Ca^2+^ regulation, signaling, and ATP production (Nita et al., 2015; Ben-Kasus Nissim et al., 2017).

Na^+^ signaling might also feed back onto astrocyte metabolism and function through a secondary regulation of ATP-sensitive K^+^-channels (K_ATP_). These channels open, promoting efflux of K^+^, when intracellular ATP concentrations decrease – as is the case following increases in [Na^+^]_i_ as described above. K_ATP_ channel expression in astrocytes is well documented (Verkhratsky and Nedergaard, 2018), where they apparently play a protective role in neurodegenerative diseases and in ischemia (Sun et al., 2008; Griffith et al., 2016; Zhong et al., 2019).

### “Atypical” Roles

While Na^+^ transients in astrocytes have abundant functional consequences due to the central role of the inward Na^+^ gradient, there are also direct effects of Na^+^ binding to diverse proteins (Figure 3). In contrast to the two prototypical ions involved in cellular signaling, Ca^2+^ and protons, endogenous buffer systems for Na^+^ are apparently absent and [Na^+^]_i_ is kept in the mM (not nM) range. Nonetheless, specific Na^+^-binding to different proteins was reported that renders their function sensitive to changes in extracellular and/or intracellular [Na^+^]. Moreover, the existence of Na^+^-responsive elements regulating an entire battery of early genes was postulated. Many of these atypical actions of Na^+^ were not yet described in astrocytes, but, in the context of this review, will be at least briefly mentioned.

#### Na^+^-Regulated Ion Channels

##### Voltage-Dependent K^+^-Channels

Na^+^ has been reported to inhibit voltage-gated K^+^ currents in various neuronal preparations, as well as in several types of glial cells (e. g., Van Damme et al., 2002). The amplitude of single-channel currents of two types of delayed rectifier outward K^+^ channels was reduced by 30 mM internal [Na^+^] in cultured Schwann cells, suggesting a channel block from the intracellular side (Howe et al., 1992). In 1994, it was shown that activation of AMPA/kainate receptors in “complex glial cells” of mouse CA1 area (according to today’s nomenclature presumably to be classified as NG2 cells) resulted in a Ca^2+^-independent inhibition of the transient component of the outwardly rectifying K^+^ current (Jabs et al., 1994). In oligodendrocyte precursor cells from embryonic cortex, Na^+^ influx through AMPA/kainate receptors blocked delayed outwardly rectifying K^+^ currents at concentrations >30 mM (Borges and Kettenmann, 1995). A similar phenomenon was described from stellate cortical astrocytes in culture. Here, opening of AMPA receptors resulted in a Na^+^-dependent block of two types of outwardly rectifying K^+^ currents that occurred at [Na^+^]_i_ between 20 and 40 mM (Robert and Magistretti, 1997).

The functional relevance and consequences of a Na^+^-dependent block of outward K^+^ currents in glial cells is only partly clear. It might prevent the release of K^+^ during periods of strong neuronal activity (and neuronal glutamate release; Robert and Magistretti, 1997). At the same time, the K^+^-current inhibition by Na^+^ will promote glutamate-induced depolarizations, an effect which will reduce glial glutamate uptake, likely aggravating excitotoxic damage under ischemic conditions. In oligodendrocyte precursor cells, there is clear evidence for an anti-proliferative role. In O-2A cells cultured from rat cortex, activation of ionotropic glutamate receptors inhibited cell proliferation. This was mediated by an increase in [Na^+^]_i_ and an ensuing block of outward K^+^ currents (Gallo et al., 1996; Knutson et al., 1997). Later, a specific role for glutamate-receptor induced inhibition of voltage-gated K^+^ channels in oligodendrocyte (but not astrocyte) development was demonstrated in organotypic cerebellar slice cultures (Yuan et al., 1998).

In addition to inhibition of outward rectifier K^+^ channels by internal Na^+^, a block of inwardly rectifying K^+^ channels (Kir) by extracellular Na^+^ was reported in cultured astrocytes (Ransom et al., 1996). Moreover, a block of Kir-channels (mainly composed of Kir2.x subtypes) by Na^+^ influx through AMPA receptors in complex glial cells was demonstrated (Schroder et al., 2002). Again, the functional significance of this is still largely unclear. Under conditions of enhanced glutamate release and accumulation in the ECS, an increase in [Na^+^]_i_ and a Na^+^-dependent reduction in glial Kir conductance might decrease their contribution to the clearance of extracellular K^+^ and thereby most likely aggravate excitotoxic conditions.

##### GIRK Channels

G-protein-gated inward rectifier K^+^ channels (GIRK) are activated by Na^+^ binding at their cytoplasmic side (Sui et al., 1996; Ho and Murrell-Lagnado, 1999). The binding of Na^+^ appears to foster the interaction of GIRK channels with phosphatidylinositol 4,5bisphosphate (PIP_2_), which is required for channel activation (Ho and Murrell-Lagnado, 1999; Yakubovich et al., 2005). The EC_50_ values reported for different GIRK channel compositions are between 27 and 44 mM Na^+^ (Ho and Murrell-Lagnado, 1999). The latter suggests their modulation by intracellular Na^+^ signaling under physiological, as well as pathophysiological conditions. Such an effect was proposed to support the inhibitory effect of vagus nerve stimulation on the heat rate following an acetylcholine-induced activation of GIRK channels in atrial pacemaker cells (Wang et al., 2014). GIRK channels are also highly expressed in the brain, where they, e. g., mediate the inhibitory action of GABA_B_ receptors in neurons (Lesage et al., 1994; Lujan and Aguado, 2015). Evidence for expression of GIRK channels (Kir3.1/Kir3.2) in astrocytes is, however, weak (Verkhratsky and Nedergaard, 2018). In retinal Müller glia cells, mRNA for both Kir3.1 and Kir3.2 was detected (Raap et al., 2002) and immunoreactivity for Kir3.1 was found in cultured astrocytes from rat cortex and spinal cord, as well as in glioma cells (Olsen and Sontheimer, 2004). The latter work thus indicates that GIRK channels – and their Na^+^-dependent regulation – might play a functional role at least in some subtypes of glial cells.

##### Na^+^-Dependent K^+^ Channels

Expression of this class of channels has to our knowledge so far not been described in astrocytes, but they shall nonetheless at least be mentioned here because of their relevance in neurons (Egan et al., 1992; Dryer, 1994). K_Na_ channels are most often composed of two subunits, called slack (or K_Na_1.1) and slick (or K_Na_1.2; Bhattacharjee and Kaczmarek, 2005). Their apparent function is to detect activity-related increases in [Na^+^]_i_, e.g., during action potential firing, upon which they open, thereby contributing to adaptation of the firing rate and to afterhyperpolarisations following bursts (Bhattacharjee and Kaczmarek, 2005).

##### Ionotropic Glutamate Receptors

Native and recombinant kainate receptors are gated by external Na^+^ (Paternain et al., 2003). In astrocytes, kainate receptors were so far only demonstrated based on mRNA and protein expression studies (Verkhratsky and Nedergaard, 2018).

NMDA receptors are expressed by neocortical (but not hippocampal) astrocytes (Schipke et al., 2001; Zhou and Kimelberg, 2001; Matthias et al., 2003; Lalo et al., 2006; Dzamba et al., 2013), and their activation results in considerable Na^+^ influx into these cells, promoting a reversal of the NCX and prolonging Ca^2+^ signals (Ziemens et al., 2019). In mammalian neurons, moderate elevation of [Na^+^]_i_ to 30–40 mM increases the open probability of NMDA receptors through a channel-associated Src kinase (Yu and Salter, 1998, 1999). The latter plays a role in the induction of long-term potentiation (LTP) at CA3-CA1 synapses of the hippocampus (Lu et al., 1998). A Na^+^-dependent regulation of NMDA receptor activity through Src kinases was also demonstrated in mouse cerebrocortical neurons. The resulting enhancement of NMDA receptor function promoted neurite outgrowth and synaptogenesis (George et al., 2012). So far, a possible role of a Na^+^-dependent regulation of NMDA receptor current in astrocytes is unexplored.

##### Volume-Regulated Anion Channels

Depending on the osmolarity of the surrounding extracellular fluids, astrocytes show adaptive regulation in the form of a regulatory volume increase (RVI) or a regulatory volume decrease (RVD; Wilson and Mongin, 2018). In the latter, volume-regulated anion channels (VRACs) are thought to play a prominent role (Kimelberg et al., 2006). In cultured rat cortical astrocytes, VRAC conductance is negatively regulated by an increase in [Na^+^]_i_ to 50 mM (Minieri et al., 2014). Based on these observations, the authors suggested that a dampening of astrocytic Na^+^ loading in ischemic conditions could present a new therapeutic target to reduce the development of brain edema.

##### Na^+^_x_ Channels

These special channels are expressed by astrocytes in the subfornical organ (SFO), which is part of the circumventricular organs and is involved in regulation of salt levels (Iadecola, 2007; Noda and Hiyama, 2015). Na_x_ channels on astrocytes are activated by an increase in extracellular [Na^+^] above 140 mM (Noda and Hiyama, 2015). This mediates a direct, Na^+^-triggered influx of Na^+^, which activates the NKA, stimulates glycolysis, and results in an extensive production of lactate (Shimizu et al., 2007). Lactate is then released by the astrocytes and taken up by neighboring GABAergic neurons, where it is used for the generation of ATP. The resulting increase in neuronal ATP levels acts on K_ATP_ channels, thereby causing an increase in their firing rate. This regulates other efferent neurons of the SFO that are involved in the behavioral control of salt intake, promoting salt aversive behavior and Na^+^ secretion (Noda and Hiyama, 2015).

#### Na^+^-Dependent Regulation of Transcription, G-Protein Signaling and Enzymes

##### Transcriptional Regulation

The group of S. Orlov was instrumental in providing evidence for an involvement of Na^+^ in gene expression (Klimanova et al., 2019). Most experiments were performed in rat smooth muscle cells, showing that an increase in [Na^+^]_i_, as, e.g., induced by application of ouabain, results in increased RNA synthesis and protein synthesis (Orlov et al., 2001). These effects were explained to result from a Na^+^-dependent activation of early response genes such as c-Fos and c-Jun, and most likely based on dedicated Na^+^-dependent response elements in those genes (Taurin et al., 2002; Haloui et al., 2007; Klimanova et al., 2019). Notably, a significant number of Na^+^/K^+^-dependent gene transcripts were also found in several other cell types, including rat brain neurons (Koltsova et al., 2012; Klimanova et al., 2019; Smolyaninova et al., 2019). The latter suggests that such regulation of gene transcription by intracellular Na^+^ might also be effective in astrocytes.

##### G-Protein-Coupled Receptors

Many class A G-protein-coupled receptors (GPCRs), including A_1_/A_2A_ adenosine and β-adrenergic receptors, as well as dopaminergic and histaminergic or mu-opioid GPCRs, show a negative modulation by Na^+^ in the physiological range (Katritch et al., 2014). Na^+^, binding to a highly conserved allosteric binding site, acts as a universal inverse agonist in these receptors; it reduces both their constitutive activity and decreases their agonist binding (Strasser et al., 2015; Zarzycka et al., 2019). Most work based on crystal structures and molecular simulations indicates that Na^+^ accesses this site from the extracellular space (Strasser et al., 2015), but for there are also simulations predicting that it can bind from the intracellular side (Selvam et al., 2018). Reported binding affinities vary widely between different receptor subtypes, but many receptors are <50 mM, suggesting a saturation with Na^+^ at a typical extracellular [Na^+^] of 145–150 mM. However, some receptors like the D4 or D2 dopamine or H1 histamine receptors show a rather low Na^+^ affinity (K_B_ >100 mM), and might, therefore, be functionally altered by physiological or pathophysiological fluctuations in extracellular [Na^+^] (Zarzycka et al., 2019).

Despite manifold evidence for Na^+^-dependent modulation of GPCRs also in the nervous system (e.g., Makman et al., 1982; Livingston and Traynor, 2014), its relevance for brain function is far from understood. In many studies, the Na^+^-binding site of GPCRs is studied in the context of serving as a possible therapeutic target (Livingston and Traynor, 2014). Mutations in the allosteric Na^+^ binding site of the orphan GPCR3, which promotes the processing of amyloid precursor protein to Aß peptides in neurons, resulted in a significant decline in Aß production, indicating that it may serve to counteract Aß accumulation in Alzheimer’s disease (Capaldi et al., 2018). Because of the widespread expression of GCPRs in glial cells (Verkhratsky and Nedergaard, 2018), it is likely that such Na^+^-dependent modulation is also relevant for astrocyte function.

##### G-Proteins

Na^+^ also affects trimeric G-proteins. In *Xenopus* oocytes, internal Na^+^ promotes the dissociation of heterotrimeric G-proteins to Gα and Gβγ (Rishal et al., 2003). This was dose-dependent with a half-maximal effect at 14 mM Na^+^ and was directly affected the Gβγ-mediated activation of GIRK channels, suggesting that Na^+^ may serve a signaling purpose (Rishal et al., 2003). In hippocampal neurons, the dissociation of Gβγ was enhanced by NMDA-receptor-mediated Na^+^ influx and resulted in the inhibition of voltage-gated Ca^2+^-channels (Blumenstein et al., 2004), again assigning Na^+^ the role of a second messenger.

Furthermore, a separate sub-class of cation-dependent G-proteins does exist, which are either K^+^-selective or activated by both K^+^ and Na^+^. The latter include different GTPases of the dynamin family which are involved in remodeling of membranes (Ash et al., 2012). The relevance of these Na^+^-dependent proteins for astrocyte function remains so far essentially unexplored.

##### Enzymes

Several enzymes are directly regulated by Na^+^. Among those are serine proteases involved in blood coagulation and complement like thrombin, which exhibits an allosteric enhancement by extracellular binding of Na^+^ that strongly enhances its catalytic properties (Dang and Di Cera, 1996; Silva et al., 2006). A recent example of an enzyme directly regulated by intracellular Na^+^ was found in skeletal muscle. Here, a member of the superfamily of calpains, namely p94/calpain3, was shown to be activated by Na^+^ at physiological concentrations, suggesting that changes in [Na^+^]_i_ during muscle contractions might be involved in the regulation of this and also other Ca^2+^-dependent enzymes (Ono et al., 2010).

Glutamine synthase is exclusively expressed by astrocytes and a key enzyme of the glutamate-glutamine cycle (Martinez-Hernandez et al., 1977; Danbolt, 2001). Moreover, it is essential in the detoxification of ammonium (NH_4_^+^), thereby protecting the brain from a harmful elevation of NH_3_/NH_4_^+^ (Albrecht et al., 2010). In 1987, Benjamin showed that several manipulations to increase [Na^+^]_i_ in rat brain tissue slices resulted in an inhibition of glutamine formation (Benjamin, 1987). He speculated that this was either related *via* a direct effect on the enzyme itself or to an accompanying decrease of ATP levels in astrocytes.

## Functional Consequences of Na^+^ Signaling in the Diseased Brain

In the previous sections, we discussed the implications of physiological Na^+^ signaling. The subsequent part will address how these functions are disrupted in response to different pathologies and what future research questions may arise from these findings. The field is only slowly moving forward in realizing the importance of Na^+^ signaling and its downstream effects, and most of the discussed work in the following sections addresses the more conventional functions of Na^+^-dependent processes in the diseased brain. Here, we mainly focus on ischemia, migraine, aging, and neurodegenerative diseases, as most studies investigating Na^+^ changes have been performed in these disease models.

### Ischemia

Stroke represents one of the leading causes of death and disability (Nedergaard and Dirnagl, 2005). As the brain is in constant need of steady oxygen and glucose supply, the abrupt reduction of cerebral blood flow (CBF) during a stroke results in the fast consumption of the remaining energy sources, pushing the regions that are particularly affected into severe energy deficits. In the core, these disruptions in the core region of ischemic stroke cause a breakdown of ionic gradients that eventually lead to cell death if not reversed (Dirnagl et al., 1999; Moskowitz et al., 2010). In the penumbra, i.e., the tissue surrounding the core, a basal level of perfusion is maintained through vascular collaterals, enhancing the chances for neuronal survival (Dirnagl et al., 1999, Moskowitz et al., 2010).

Two factors can negatively affect the outcome of survival in the penumbra: (1) the available energy is too low for glutamate transporters to efficiently clear glutamate from the synaptic cleft, resulting in excess extracellular glutamate and intracellular Ca^2+^ leading to excitotoxicity (Rakers and Petzold, 2017) and (2) peri-infarct depolarizations (PID), i.e., repeated waves of spreading depolarizations traveling through the penumbra that lead to more glutamate release and an even higher energy consumption (Hossmann, 1996; Lauritzen et al., 2011; Rakers and Petzold, 2017). Astrocytes play an important role in the reduction of PID-induced excitotoxic mechanisms, owing to their ability to remove glutamate from the synaptic cleft (Nedergaard and Dirnagl, 2005). This uptake of glutamate during PIDs into astrocytes by EAATs produces an increase in their [Na^+^]_i_, a process aggravated by reduced NKA activity due to energy shortage in the hypoxic tissue (Rose and Karus, 2013).

The resulting strong elevation of [Na^+^]_i_ in neurons and astrocytes has several important consequences for cell function and survival in the ischemic brain. First, the scarce ATP resources that are left in the penumbra are further reduced by cells attempting to normalize Na^+^ and K^+^ homoeostasis through the NKA. Second, the failure to fully reconstitute Na^+^/K^+^ homoeostasis further aggravates extracellular K^+^ accumulation, promoting depolarization and thus the initiation of more PIDs. Third, the [Na^+^]_i_ increase reduces the driving force for glutamate uptake, further increasing extracellular glutamate half-life and thereby excitoxicity (Rossi et al., 2000). Finally, Na^+^ accumulation in neurons and astrocytes leads to a cellular overload with Ca^2+^ through reverse action of the NCX. This is because, as intracellular Na^+^ levels become exceedingly high in the ischemic penumbra due to the abovementioned mechanisms, NCX reverses its action to shuttle excess Na^+^ out of the cells in exchange for more Ca^2+^, contributing to Ca^2+^ overload.

**Figure 4:**
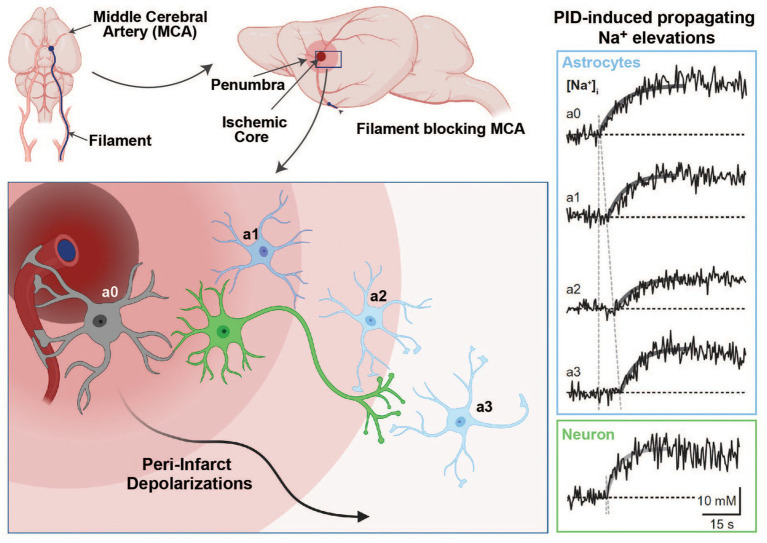
Na^+^ signaling in the experimental middle cerebral artery occlusion (MCAO) stroke model. Induction of an ischemic stroke by insertion of a filament into the internal carotid artery until the MCA is blocked **(upper panel)**. In the ischemic core, dying cells initiate peri-infarct depolarizations (PIDs), which travel through the penumbra and lead to excess glutamate release and higher energy consumption **(bottom left panel)**. Glutamate uptake by astrocytes is accompanied by Na^+^ cotransport through glutamate transporters, which results in detectable intracellular Na^+^ elevations in response to PIDs (**right panel**; Na^+^ traces adapted with permission from Gerkau et al. 2018; images created with BioRender.com).

In a recent study, we addressed these mechanisms experimentally during PIDs *in vivo* and *in vitro* (Gerkau et al., 2018). We used multiphoton and wide-field Na^+^ and Ca^2+^ imaging in the *in vivo* rodent stroke model of middle cerebral artery occlusion (MCAO) and in tissue slices (Gerkau et al., 2018). As expected, we found an association between PIDs and propagating Na^+^ elevations in neurons and astrocytes *in vivo*, as well as in tissue slices that underwent brief chemical ischemia (Figure 4). The blockade of NMDA-receptors reduced PID-related Na^+^ and Ca^2+^ elevations in both cell types. The pharmacological inhibition of NCX revealed a strong reduction in ischemia-induced intracellular Ca^2+^ signaling in neurons and astrocytes and led to an increase in [Na^+^]_i_ in both cell types (Gerkau et al., 2018). Thus, these results provided direct experimental evidence that the reversal of NCX during metabolic failure is a major source of cellular Ca^2+^ increases in neurons and astrocytes while dampening their Na^+^ overload.

The role of NCX in ischemic damage was also investigated by other studies that used knockout strategies to target different NCX isoforms. The first study used two lines of complete knockout animals deficient for NCX2 and NCX3, respectively, and evaluated the effect of the deletion after inducing an ischemic stroke by MCAO (Molinaro et al., 2013). Both KO lines showed increased neuronal vulnerability and increased infarct sizes. In the second study, the authors conditionally overexpressed or deleted the NCX1 isoform in cortical and hippocampal neurons and investigated its influence on stroke outcome (Molinaro et al., 2016). The conditional deletion of neuronal NCX1 led to increased brain damage and neurological deficits, while the overexpression of NCX1 resulted in reduced ischemic volume and an amelioration of neuronal deficits. These findings are surprising, given that, presuming a reverse mode of NCX, a neuron-specific deletion of NCX1 should lead to a lower calcium concentration and less neurotoxicity in the penumbra in neurons. In line with this notion, pharmacological inhibition of NCX revealed a strong reduction in ischemia-induced intracellular Ca^2+^ levels in neurons during ischemia, and PIDs, in particular, and should therefore lead to a better outcome (Gerkau et al., 2018). On the other hand, a neuron-specific deletion of NCX (as used in Molinaro et al., 2016) should also lead to increased intracellular and decreased extracellular Na^+^ levels under reverse-mode conditions, perhaps further decreasing the driving force for glutamate uptake into astrocytes. Therefore, it will be important to also directly measure membrane potentials, as well as glutamate, Na^+^, and Ca^2+^ levels in neurons and astrocytes *in vivo* during different levels of ischemia to address these open questions (Rakers and Petzold, 2017). Likewise, the cell-specific role of NCX remains to be established. For example, a deletion of NCX in astrocytes should result in lower astroglial Ca^2+^ levels during ischemia, and this may lead to a decreased accumulation of glutamate due to less Ca^2+^-dependent glutamate release by astrocytes. On the other hand, it may also decrease the driving force for Na^+^-glutamate-cotransport during PIDs, so the net effect on glutamate is unclear.

### Migraine

Familial hemiplegic migraine 2 (FHM2), which is a rare autosomal-dominant subtype of migraine with aura, is linked to a loss-of-function mutation in the α2 subunit of NKA (De Fusco et al., 2003). Migraine aura is induced by cortical spreading depressions (CSD), and accordingly, CSD induction is facilitated in heterozygous FHM2 knock-in animals carrying this mutation. Capuani et al. (2016) showed 50% less NKA expression and a reduction of GLT-1a in astrocytic processes at cortical glutamatergic synapses in the dentate gyrus (DG) of these mice. This caused a reduced glutamate and K^+^ clearance by astrocytes in cortical acute slices (Capuani et al., 2016), indicating that impaired glutamate clearance mechanisms may lead to the facilitation of CSDs. A recent study using the same model investigated synaptic plasticity processes in the hippocampus and found an abnormally increased long-term potentiation (LTP) in the dentate gyrus (DG), while CA1 LTP was not changed compared to wild type (WT) animals (Iure et al., 2019). Interestingly, basal synaptic transmission in these animals was unaltered in all investigated regions, pointing to an activity-dependent effect on synaptic plasticity processes. The previously described reduction in GLT-1a expression that is correlated to the reduced NKA α2-subunit expression may, thus, result in decreased glutamate clearance leading to the abnormally high LTP.

Of particular interest in this context is the finding that GLT-1 is not ubiquitously expressed at the same level within the hippocampus, but shows higher levels in the CA3 and DG, while in CA1 its expression is lower (Furuta et al., 1997), correlating with the electrophysiological LTP data. Another study investigated the impact of the heterozygous deletion of the NKA α2-subunit on behavior and reported increased anxiety-related behavior, reduced locomotor activity, and impaired spatial learning in the Morris water maze in these mice (Moseley et al., 2007). Thus, the reduction in α2 NKA activity either in the FHM2 loss-of-function mutation or heterozygous deletion of that subunit could play a crucial role in the observed memory deficits in patients with this type of migraine (Karner et al., 2012). Thus, the reduced NKA activity leads to dysregulated glutamate clearance, resulting in abnormally increased long-term synaptic plasticity, which may impact memory functions in patients (Dilekoz et al., 2015).

### Aging

During aging, NKA activity declines in the CNS, but protein levels stay unchanged, pointing to an age-dependent downregulation of its activity (Scavone et al., 2000; Kawamoto et al., 2008; Vasconcelos et al., 2015). The decrease in NKA activity can lead to an increase in intracellular Ca^2+^, most likely by the abovementioned downstream activation of reverse-mode NCX, which in turn may elicit excitotoxicity (Mattson and Liu, 2002). Kawamoto et al. (2008) found that the decreased NKA activity is also linked to changes in glutamate transport pathways, which again may lead to disturbances in the ionic homeostasis that may, if chronically perturbed, predispose to the development of neurodegenerative diseases (Kinoshita et al., 2016).

Mitochondria represent another important factor that can become compromised during aging and thereby increase the likelihood to develop a neurodegenerative disorder (Yankner et al., 2008). Impaired mitochondrial energy supply can directly affect NKA activity by producing less ATP, but also by production of more free radicals (Nathanson et al., 1995). Several studies showed that the NKA α subunit is sensitive to oxidative stress by free radicals, showing a degradation of the subunit (for review see Kinoshita et al., 2016).

A reduction of NKA activity by these different age-dependent mechanisms may result in a reduction in the Na^+^ gradient that is crucial for the proper functioning of glutamate clearance mechanism after neuronal firing under healthy conditions. In this context, it has been shown that NKA impairment leads to a downregulation of synaptic AMPA receptors, which results in defective synaptic transmission and cognitive decline as a consequence (Zhang et al., 2009).

In addition to changes in glutamate receptor expression, the levels of EAAT glutamate transporters have been described to decrease in the cause of many neurodegenerative diseases (Sheldon and Robinson, 2007). As discussed above for the FHM2 model, the reduced capacity to remove glutamate out of the synaptic cleft after neuronal activity will lead to excitotoxicity, which will lead to further disruptions in NKA activity, thereby initiating a vicious circle.

### Neurodegenerative Diseases

Alzheimer’s disease (AD) is the most prevalent form of dementia and is characterized by memory loss and cognitive impairment. Its two pathological hallmarks are the extracellular deposition of amyloid-β peptide in senile plaques and the intracellular aggregation of hyperphosphorylated tau fibrils in neurofibrillary tangles (Hardy, 2006). Several studies have revealed that these proteopathic changes are also accompanied by altered NKA activity and a disturbed Na^+^ homeostasis. An *in vitro* study showed that the acute application of Aβ oligomers to rat hippocampal neurons reduced NKA activity (Mark et al., 1995). This impairment resulted in an accumulation of intracellular Na^+^ and successive influx of Ca^2+^, pointing to an important role of the NKA in pathological dysregulation in AD (Mark et al., 1995). More recently, a decrease in hippocampal NKA activity, as well as a reduction in overall protein levels, was demonstrated in an AD mouse model (Dickey et al., 2005). The latter studies did not further differentiate between neuronal or glial NKA levels or activity. Others, however, found that the *in vitro* treatment of primary astrocytes with Aβ oligomers resulted in a downregulation of NKA and an imbalance of Na^+^ and K^+^ concentrations, an effect replicated in postmortem tissue from AD patients (Vitvitsky et al., 2012; Graham et al., 2015). As the NKA maintains the electrochemical Na^+^ gradient that provides the main driving force for glutamate uptake by EAATs, this may in part explain changes observed in extracellular glutamate half-life in the vicinity of amyloid plaques (Hefendehl et al., 2016).

About 20% of familial forms of amyotrophic lateral sclerosis (ALS) are characterized by mutations in the superoxide dismutase 1 (SOD1) gene. One study found an enrichment of a protein complex consisting of α2-NKA and α-adducin in astrocytes of SOD1^G93A^ model mice. The knockdown of both proteins in astrocytes or the pharmacological blockade of the NKA with digoxin prevented motor neuron degeneration (Gallardo et al., 2014). Interestingly, the same study also reported an upregulation of both proteins in spinal cord lysates of ALS patients. This suggested that chronic activation of the α2-NKA/α-adducin complex might represent a critical pathological mechanism for motor neuron degeneration that could serve as a potential target in this disease. Of note, mechanisms independent of NKA leading to ion disequilibrium in ALS have been described as well (Almad et al., 2016). Future studies will be necessary to reveal the impact of these changes on Na^+^ homeostasis and regulation and glutamate levels.

## Conclusions

Astrocytes are the main supportive cells of the CNS playing important homeostatic roles. Many of the astroglial regulatory processes that are initiated in response to neuronal activity are directly correlated to transient changes in their [Na^+^]_i_, which were demonstrated to occur in various preparations and brain regions. While it is widely accepted that astrocyte [Na^+^]_i_ indeed changes with different forms of neuronal activity, many highly relevant questions as to the functional consequences of such fluctuations in Na^+^ still remain unanswered.

Of foremost importance is to further differentiate and thereby clarify the specific physiological functions of astrocytic Na^+^-dependent signaling pathways and to separate them from neuronal ones. The central player mediating Na^+^ efflux and controlling the distribution of all major ions in both cell types is the NKA, both by directly controlling the levels of Na^+^ and K^+^ and indirectly by affecting the transport of Ca^2+^ (*via* NCX), Cl^−^ (*via* NKCC1), and protons (*via* NHE or NBC). In addition, alteration of astrocytic Na^+^ levels can have direct effects on glial signaling, as well as on neuronal properties, by changing the driving force for transmitter transporters. However, more studies addressing glia-specific NKA regulation, its relation to astroglial [Na^+^]_i_ and Na^+^ signals, and the functional consequences of the latter, are needed. The same is true for most, if not all, other processes involving Na^+^ transport across the plasma membrane and generating [Na^+^]_i_ changes in astrocytes.

Besides the direct effect of [Na^+^]_i_ on transporters and channels, there is increasing evidence that Na^+^ can interact with several other binding partners. The latter include ion channels, GPCRs, trimeric G-proteins, or enzymes. By modulating the functions of these interacting proteins, changes in [Na^+^]_i_ can alter the physiological state of the cells. Furthermore, there is evidence that Na^+^ is directly involved in gene regulation through Na^+^-responsive elements, affecting the expression of early response genes. These atypical modulatory functions of Na^+^ have mainly been investigated in cell types other than astrocytes; therefore, more experimental work needs to be performed to study their role in astrocytes. Such work could significantly impact our understanding of various CNS pathologies, where changes in several of the atypical Na^+^-interacting partners have been described, but a possible direct link to Na^+^ signals and especially Na^+^ signals in astrocytes has not yet not been addressed.

Due to the technical progress in the field, our knowledge of astrocyte physiology and Na^+^ signaling in particular has increased tremendously within the last 2 decades. These advances have revealed a role for Na^+^ far more active than only offering a convenient, transporter-driving gradient. However, our understanding of its putative role as an intracellular messenger system and the extent to which it interacts with partners in astrocytes is far from complete. Until now, tools to study Na^+^ within cells have been very limited in comparison to those available for Ca^2+^. However, the development of techniques such as fluorescent lifetime imaging and genetic reporter animals could help elucidate the true importance of astrocytic Na^+^ homeostasis under physiological as well as pathological conditions.

## Author Contributions

All authors contributed to the design, concept, and writing of this review. All authors contributed to the article and approved the submitted version.

### Conflict of Interest

The authors declare that the research was conducted in the absence of any commercial or financial relationships that could be construed as a potential conflict of interest.
